# Helminth infection in southern Laos: high prevalence and low awareness

**DOI:** 10.1186/1756-3305-6-328

**Published:** 2013-11-14

**Authors:** Khampheng Phongluxa, Vilavanh Xayaseng, Youthanavanh Vonghachack, Kongsap Akkhavong, Peter van Eeuwijk, Peter Odermatt

**Affiliations:** 1 National Institute of Public Health, Ministry of Health, Vientiane, Lao PDR; 2 Department of Epidemiology and Public Health, Swiss Tropical and Public Health Institute, P.O. Box, CH-4002, Basel, Switzerland; 3 University of Basel, Basel, Switzerland; 4 Institute of Social Anthropology, Basel, Switzerland; 5 University of Health Science, Ministry of Health, Vientiane, Lao PDR

**Keywords:** Helminth infection, Soil-transmitted helminth, Liver fluke, Raw fish consumption, Latrine use, Health education

## Abstract

**Background:**

Helminthiasis is a public health concern in Lao People’s Democratic Republic (Lao PDR, Laos). This study aimed to understand helminth infection and associated risk factors in relation to existing local knowledge, attitudes and practices regarding worm infections in endemic communities.

**Methods:**

A cross-sectional survey was conducted in 10 randomly selected villages in Saravane district, Southern Laos. Two stool samples obtained from 574 members (aged ≥2 years) of selected households were examined using the Kato Katz method. Household heads (n = 130) were interviewed. Eight focus group discussions (FGDs) and direct observations were performed. Uni- and multivariate logistic regression analyses were conducted to predict infection. Content analysis was conducted for qualitative data.

**Results:**

The prevalence of *Opisthorchis viverrini*, hookworm, *Trichuris trichiura, Ascaris lumbricoides* and *Taenia* sp. was 88.7%, 86.6%, 32.9%, 9.8% and 11.5%, respectively. Most individuals were co-infected with *O. viverrini* and hookworm. More men had multiple helminth infections than did women. Only one-third of household heads had heard about liver fluke before, of which 59.2% associated it with eating raw fish dish. Among the soil-transmitted helminths, roundworm was the most well known (70.8%) but was attributed to raw food consumption (91.3%). Eating raw fish was a common practice (75.4%); few households possessed a latrine (16.1%); less than half of the study participants mentioned health benefits from latrine use and personal hygiene. Focus group discussion participants had a low level of awareness of worm infections; more men liked eating raw fish than did women; some disliked using latrines because they were not used to it and because of their bad smell. Poor personal and village hygiene practices were observed.

**Conclusions:**

This study highlights a high helminth infection rate and poor community awareness of worm infections and associated risk factors. Only a sound awareness of worm infection and the underlying risk factors may prevent infection and re-infection after treatment.

## Background

Food-borne trematodiases (FBT) are among the neglected tropical diseases [1] and a public health problem in many parts of the world, with a global burden of 665,000 disability adjusted life years [2]. FBT often co-exists with soil-transmitted helminthiasis (STH) [3,4]. About 750 million people are at risk for FBT, of which 40 million are infected [5]. An estimated 1.2 billion, 800 million and 740 million people worldwide are infected with *Ascaris lumbricoides*, *Trichuris trichiura*, and hookworm, respectively [6]. In Southeast Asia, about 67.3 million people are at risk for *Opisthorchis viverrini,* the most frequently observed FBT*.* Ten million people are infected in Thailand and Laos [7]. *O. viverrini* infection is common where consumption of raw or insufficiently cooked fish dishes is deeply rooted in local culture and where proper sanitation is minimal or absent [1]. *O. viverrini* infection results in hepatobiliary morbidity [2] and chronic infection may lead to cholangiocarcinoma, a fatal bile duct cancer [8]. Preventive chemotherapy together with health education is the most current helminth control strategy [9]– [11]. However, re-infection rates after treatment with anti-helminthic drugs are rapidly reaching pre-treatment prevalence rates [12] as food and hygiene behaviour patterns remain unchanged, indicating that health education is not effectively addressing the critical issues.

Deepening our understanding of helminth infections in relation to local notions of these infections, its risk factors and deworming measures is important for developing sustainable and effective helminth control. For example, in northern Vietnam, local communities were aware of the risks of eating raw fish dish, however, people were less cautious about consuming raw fish dishes because, they argued, with the effective drug, they could be easily cured after an infection [13]. Having a good knowledge of worm infection and of people’s perception of the risk factors and of the benefits of deworming facilitates the development of valid interventions and convinces people to comply with the intervention [14]– [17]. Hence, an in-depth knowledge of infection prevalence and of the risks of endemic helminths and an understanding of corresponding local knowledge and perception patterns is essential for improving helminth control activities in the local setting.

In Laos, over two million people are infected with liver fluke [7]. Recent surveys reveal a rampant *O. viverrini* infection rate of more than 90% of the general population in southern areas [18]– [20]. About 1.6 million children under 15 years of age are at risk of STHs. However, our understanding of the local knowledge, attitudes and practices in relation to worm infection and its risk factors is limited. Today, preventive chemotherapy combined with health education via vertical programmes remains the main strategy for helminth control in Laos. It is implemented without detailed knowledge of the attitudes and practices of the local communities. Therefore, underlying risk factors such as food preparation and hygiene behaviour remain inadequately addressed and, hence, re-infection rates are high.

This study aimed to advance our understanding of helminth infection in relation to the community’s existing knowledge, attitudes and practices in endemic areas in southern Laos. A cross-sectional survey was conducted in randomly selected villages and households in Saravane district to assess helminth infections and their risk factors. Focus group discussions (FGDs) and direct observations were carried out to triangulate knowledge, attitudes and practices with regard to helminth infections.

## Methods

### Ethics statement

Ethical clearance was obtained from the National Ethics Committee on Health Research, Ministry of Health, Lao PDR (Ethical Clearance No 169/NECHR, 1 April 2008). Written informed consent was obtained from heads of selected households. For illiterate persons, informed consent was read out and, after approval, the person signed with a fingerprint. The interviewees were informed about the study aim, the study procedures, the need for voluntarily participation, and their right to stop participation at any time. All infections diagnosed in this study were treated according to the national treatment guidelines [21].

### Study areas and population

**Figure 1:**
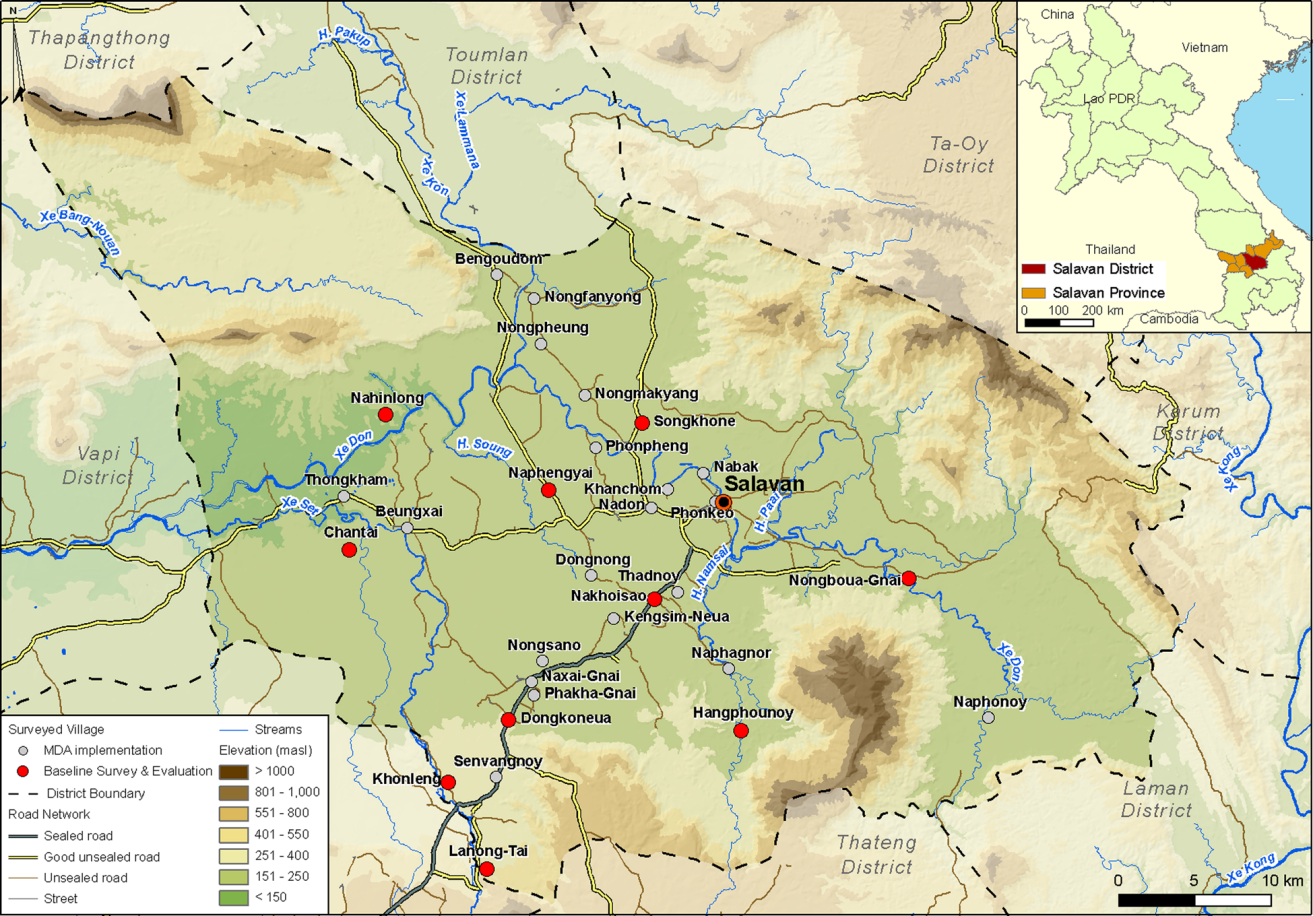
Study villages (red spots) in Saravane district, province of Saravane, Lao PDR.

The study was conducted in ten randomly selected villages of Saravane district, Saravane province (Figure 1). The total population in these villages comprised 6,207 inhabitants (including 3,056 females and 847 children under five years of age). Some villages were located near rivers, namely Xedonh and Xeseth, and their tributaries (Houay Lanong, Houay Namxai, and Houay Nongboua). In some villages, the Laotheung ethnic group formed the majority of the population. The main occupation of residents was subsistence rice farming. Daily food intake consisted of rice with vegetables and bamboo, as well as fish from rivers and ponds and game. In four villages (Nakhoisao, Nahinlong, Naphengyai, and Hangphounoy), demonstration latrines were constructed by the Saravane Provincial Health Office and international projects.

Saravane district is located on the Bolaven Plateau. It covers 2,441 km^2^ and has a population density of three people per square kilometre (km^2^). The 89,068 inhabitants (female: 45,520) live in 178 villages and in 13,239 households. Ethnic groups include the Laotheung and the Laoloum. The annual income per capita is USD 627. There are nine health centres, which cover 71% of the villages. The district has 19 pharmacies, 91 villages with drug kits, two private clinics and 13 outreach teams (Report Saravane District Health Office [DHO], 2008). In 2002, a national parasitology survey indicated that 21.5%, 11.9%, 10.9% and 5.4% of primary school children in Saravane province were infected with *O. viverrini*, *A. lumbricoides*, hookworm and *T. trichiura,* respectively [22]. In 2004, the prevalence of *O. viverrini* infection was estimated at 58.5% (village range from 20.0% to 85.5%) in Saravane district. The infection rate increased with age. Eating raw or insufficiently cooked fish dishes was very common (79.7%). Only one in 13 villages had latrines. Sixty per cent of the cyprinoid fish species consumed in this district are infected with *O. viverrini* metacercariae [23]. Mass treatment against helminth infection does not yet exist in Saravane district (Director of Saravane Provincial Health Office [PHO]).

### Study design

A cross-sectional survey was carried out in January and February 2010, using two-stage cluster sampling. First, 10 villages were randomly selected from the list of villages in the district obtained from the Saravane DHO. Second, 20 households were randomly selected from the household list. All household members aged 2 years and older were enrolled and intestinal helminth infections were diagnosed from two stools samples. Heads of households were interviewed. Eight FGDs were carried out in four randomly selected villages; in each village two FGDs were conducted: one among men and another among women. Direct observations were performed in all villages.

### Field and laboratory procedures

Village leaders and household heads were informed about the study’s objectives and procedures. A structured interview was conducted with heads of selected households, where information on socio-demographic household characteristics, household assets, and knowledge, attitudes and practices relating to *O. viverrini* and helminth infections (e.g. raw fish consumption, hygiene and sanitation behaviour) were collected. FGD participants were randomly selected from households that were not part of the cross-sectional survey. In FGDs, open-ended and open questions were used, i.e. (i) Have you heard about liver fluke/roundworm/hookworm/whipworm? (ii) If yes, could you please tell us how people get this infection? (iii) What is the relationship between infection and disease? (iv) How can you prevent it? (v) Do you eat raw fish dishes? How often? (vi) What do you think about eating raw fish dishes? (vii) Do you have a latrine? What do you think about using latrines? (viii) What are the benefits of using a latrine and of hand washing before eating/after defecation? (ix) What do you think about hand washing before eating and after defecation? (x) Do you wash your hands before eating and/or after defecation? Team leaders made direct observations during visits in the communities regarding the cleanliness of the village, the consumption of raw fish dish, personal hygiene practices, wearing shoes and the presence of sanitation facilities.

In the cross-sectional study, for each enrolled household member, two stool samples were collected on two consecutive days and examined. Stool containers labelled with date, ID, name, age and sex of participant were handed-out. Heads of households were instructed on how to fill the container with stool samples. On the collection day, people who gave a first stool sample received a second pre-labelled container to use for the following day. All collected samples were kept in a cool box and were transported by car to the provincial hospital’s laboratory in Saravane within an hour after collection. For each stool sample, one Kato Katz thick smear slide was created, using standard 41.7 mg templates [24]. After a clearance time of 30 minutes, the slide was examined under a light microscope (100 × magnification). All samples were examined on the day of collection. The number of eggs per parasite was counted and recorded for each parasite species separately.

### Data management and statistical analysis

Data were entered twice (double entry) into EpiData, version 3.1 (Epidata Association; Odense, Denmark) and validated. Analysis was performed using Stata software, version 10.1 (Stata Corp., College Station, TX, USA). Only participants with two stool samples were retained in the analysis. Individuals were divided into seven age groups (in year) (<6, 6–15, 16–29, 30–39, 40–49, 50–59 and 60+); most participants were aged between 6–15 (26.8%), 16–29 (21.8%) and 30–39 (16.0%). Descriptive statistics were calculated (counts, percentages, means and standard deviations [SD]). The intensity of helminth infections was expressed in terms of egg count per gram faeces (EPG). According to Maleewong *et al*. and Sayasone *et al.*[23,25], for the infection with *O. viverrini,* and STHs the following light, moderate, and high intensity groups were established based on the EPG counts: Hookworm, 1–1999 EPG, 2000–3999 EPG, and ≥4000 EPG; *A. lumbricoides*, 1–4999 EPG, 5000–49,999 EPG, and ≥50,000 EPG; and *T. trichiura* and *O. viverrini*, 1–999 EPG, 1000–9999 EPG, and ≥10,000 EPG. Pearson’s χ^2^ and Fisher’s exact tests were used to compare proportions. The association between *O. viverrini* infection and sex, age group, ethnicity, raw fish consumption, having a latrine, and socio-economic status (SES) was assessed in a univariate logistic regression analysis. Predictors with p < 0.25 were retained in a multivariate logistic regression model. Odds ratio (OR) and 95% confidence intervals (95% CI) were reported. P-values less than 5% were considered significant.

SES of households was assessed using Multiple Correspondence Analysis (MCA). The technique has been used before for categorical data [18]. Household assets, materials used for house construction, water source and possession of a latrine were used to construct a socio-economic index. Households were classified into one of five quintiles: most poor, very poor, poor, less poor and least poor, using the socio-economic index.

Qualitative data from FGDs were transcribed from notes and tape recordings, then translated from Lao into English, typed into MSWord and imported to MAXQDA (version 10) software for textual analysis. Statements were coded and categorised according to the following themes: knowledge of liver fluke, hookworm, whipworm, roundworm and tapeworm; raw fish consumption; hand washing before eating; hand washing after latrine use and deworming. Coded data were retrieved and exported to MSExcel for frequency and content analysis.

## Results

### Characteristics of the study population

**Figure 2:**
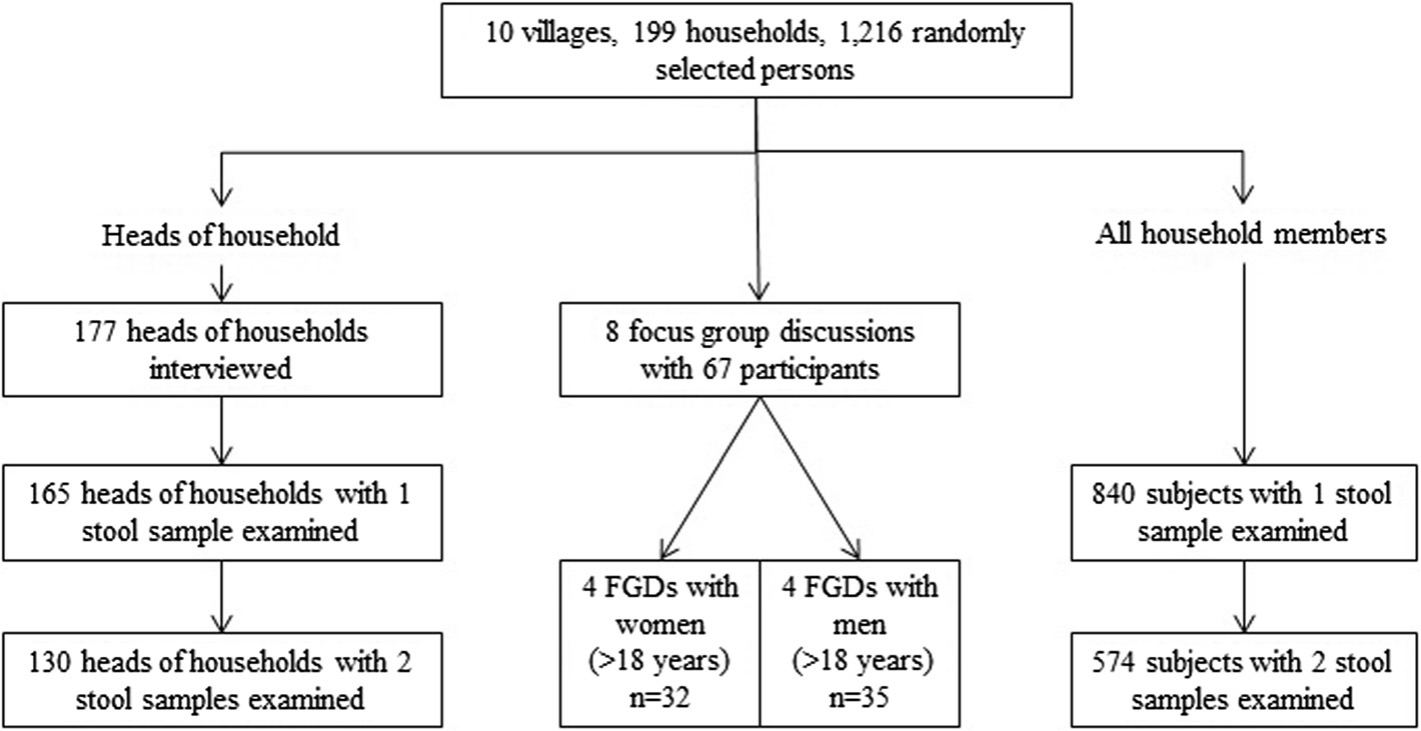
Study diagram, Saravane district, 2010.

One hundred and thirty heads of households were retained in the analysis (Figure 2); 53.1% were male; the mean age was 41.2 years (SD 13.6 years, range: 20–81 years); mean age by gender was 43.4 years (SD 14.6 years; range 20–81 years) for men and 38.5 years (SD 11.8 years; range 21–74 years) for women. Almost all household heads were farmers (93.1%); 56.9% had completed primary school while 29.2% had not attended school; 56.9% belonged to the Laoloum ethnic group.

From the enrolled households, 574 people submitted two stool samples (Figure 2); 50.3% of these were from females; the mean age was 25.5 years (SD 18.2 years, range: 2–81 years). There was no significant age difference between genders: the mean age for males was 24.4 years (SD 18.2 years, range 2–81 years, 95% CI 22.3–26.5) and 26.6 years (SD 18.3 years, range 2–74 years, 95% CI 24.5–28.7) for females; 53.3% belonged to the Laoloum ethnic group.

Among 67 FGD participants, 52.2% were females. The mean age was 40.6 years (range 15–73 years). Most of them were in the age groups of 35–44 years (32.8%) and 25–34 years (22.4%); more than two-third of the FGD participants belonged to the Laotheung ethnic group (71.6%) and were Buddhists (71.7%); 55.2% had attended primary school while 40.3% had not attended school at all. All were farmers.

### Helminth infection prevalence, intensity and risk factors

Intestinal helminth infections were highly prevalent. *O. viverrini,* hookworm, *T. trichiura* and *A. lumbricoides* were diagnosed in 88.7%, 86.6%, 32.9% and 9.8% of the individuals, respectively. In addition, *Taenia* sp. was detected in 11.5% of the study participants. *O. viverrini* infection intensity increased with age and reached a mean infection intensity of 2163 EPG in the oldest age group. *O. viverrini*, hookworm and *A. lumbricoides* infection rates were not significantly different between genders but *T. trichiura* infection was significantly more frequent in male participants than in female participants (p = 0.031, Table 1).

**Table 1:** Helminth infection prevalence and intensity by gender and age group (in years), Saravane district, 2010 (n = 574)

**Item**	**n (%)**	**Male**	**Female**	**<6**	**6-15**	**16-29**	**30-39**	**40-49**	**50-59**	**60+**
*Opisthorchis viverrini*	509 (88.7)	91.2	86.1	72.6	87.0	88.8	91.3	98.3	95.2	100
GM faecal egg count (range), EPG:	925* (24 – 69,648)	1100.3	771.5	221.6	406.0	1337.9	1388.7	2508.7	1890.7	2162.9
Intensity infection:										
Light (1–999 EPG)	261 (51.3)	47.7	55.0	88.7	70.9	45.0	36.9	28.1	30.0	33.3
Moderate (1000–9999 EPG)	184 (36.1)	38.1	34.1	9.43	23.9	40.5	48.8	49.1	50.0	43.3
Heavy (≥10000 EPG)	64 (12.6)	14.2	10.8	1.9	5.2	14.4	14.3	22.8	20.0	23.3
Hookworm	497 (86.6)	88.8	84.4	71.2	87.0	86.4	92.4	86.2	92.9	96.7
GM faecal egg count (range), EPG:	446.5* (24 – 38,880)	492.2	403.6	342.1	393.5	443.9	538.7	381.4	543.1	768.5
Intensity of infection:										
Light (1–1999 EPG)	426 (85.7)	84.6	86.9	90.4	86.6	88.9	77.6	96	79.5	75.9
Moderate (2000–3999 EPG)	33 (6.6)	8.3	4.9	3.8	7.5	3.7	10.6	0.0	15.4	6.9
Heavy (≥4000 EPG)	38 (7.7)	7.1	8.2	5.8	5.9	7.4	11.8	4.0	5.1	17.2
*Trichuris trichiura*	189 (32.9)	37.5	28.4	38.4	35.1	29.6	29.4	37.9	33.3	23.3
GM faecal egg count (range), EPG:	189.6* (24 – 10,800)	179.9	202.9	187.9	179.1	221.4	156.3	253.5	128.8	246.2
Intensity of infection:										
Light (1–999 EPG)	164 (86.8)	85.9	87.8	92.9	81.5	86.5	88.9	90.9	92.9	71.4
Moderate (1000–9999 EPG)	24 (12.7)	14.0	10.9	7.1	16.7	13.5	11.1	9.1	7.1	28.6
Heavy (≥10000 EPG)	1 (0.5)	0	1.2	0	1.8	0	0	0	0	0
*Ascaris lumbricoides*	56 (9.8)	8.1	11.4	19.2	8.4	9.6	7.6	8.6	4.8	10.0
GM faecal egg count (range), EPG:	2984.5* (96 – 178,080)	3031.2	2952.4	6566.2	6691.1	757.1	4416.9	656.1	1515.4	4321.5
Intensity of infection:										
Light (1–4999 EPG)	35 (62.7)	65.2	60.6	42.9	53.8	83.3	57.1	100	50.0	66.7
Moderate (5000–49999 EPG)	15 (26.8)	21.7	30.3	42.9	23.1	16.7	28.6	0	50.0	33.3
Heavy (≥50000 EPG)	6 (10.7)	13.0	9.1	14.3	23.1	0	14.3	0	0	0
*Taenia* sp.	66 (11.5)	11.6	11.4	6.8	5.8	13.6	14.1	15.5	21.4	13.3

GM= geometric mean; EPG= egg per gram of stool; *= mean of parasite egg.

**Figure 3:**
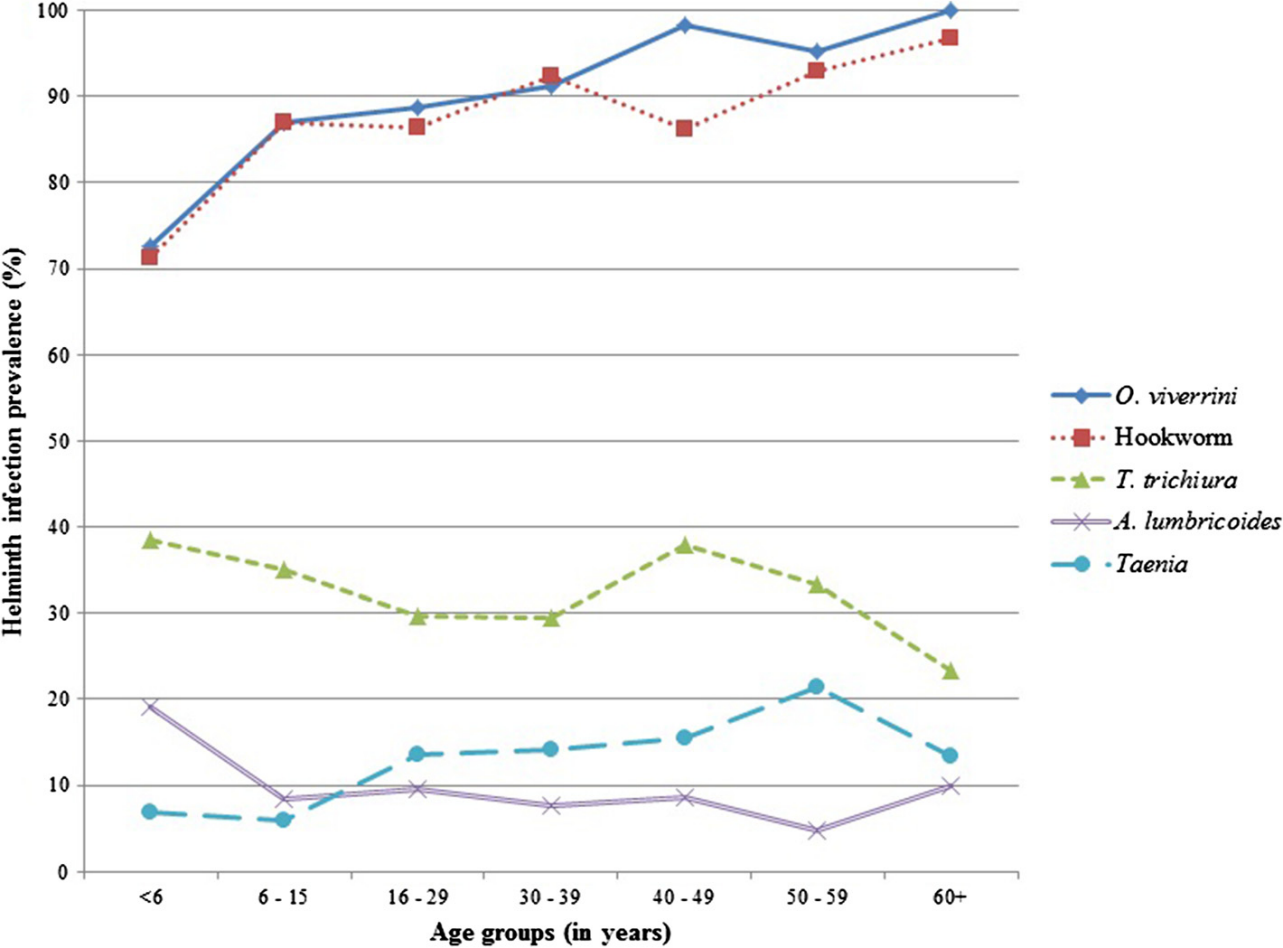
Helminth infection rate by age groups, Saravane district, 2010 (n = 574).

Prevalence of *O. viverrini* and hookworm increased significantly with age, from 72.6% to 100% (p < 0.001) and from 71.2% to 96.7% (p = 0.001) from the youngest to the oldest age group, respectively, whereas *Taenia* sp. increased from 6.8% at ages <6 years to 21.4% among people aged 50–59 and then declined to 13.3% at ages >59 (p = 0.034, Figure 3).

**Figure 4:**
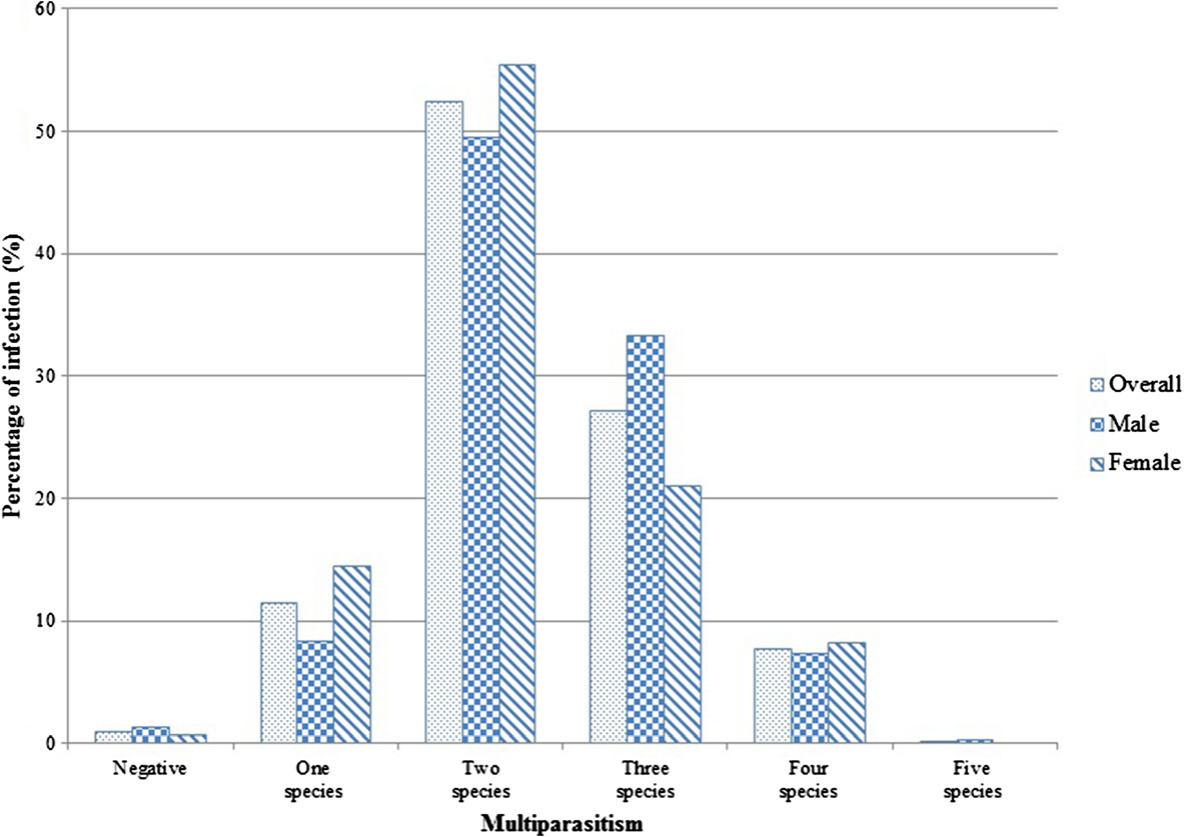
Multiple helminth infections in study population by gender in Saravane district, 2010 (n = 574).

Multiparasitism was very common. More than half of the participants (52.4%) harboured two helminth species or more. Three different species were diagnosed in 27.2% of the participants. Infection with three helminth species was detected in males significantly more (33.3%) than in females (21.1%, p = 0.005, Figure 4). Among *O. viverrini*-infected people, more than three quarters (77.3%) were co-infected with hookworm, of which 74.7% were children in the age group 6–15 years.

### Risk factors of *O. viverrini* infection

After an initial univariate prediction of *O. viverrini,* hookworm, *T. trichiura* and *A. lumbricoides* infection in all study participants, the following variables were included in the multivariable logistic regression analysis model for predicting hookworm: gender, age group, ethnicity and having a latrine; only the first three variables were included for predicting *O. viverrini* and *A. lumbricoides* and only gender and ethnicity were included for predicting *T. trichiura*. Results indicated that males had a strongly increased risk for *O. viverrini* infection compared to females (OR = 1.9, p = 0.023). Laoloum had a significantly higher risk for *O. viverrini* infection than did Laotheung (OR = 2.3, p = 0.003) but a lower risk for hookworm infection (OR = 0.59, p = 0.049), *T. trichiura* (OR = 0.25, p < 0.001) and *A. lumbricoides* (OR = 0.27, p < 0.001) compared to Laotheung. Children aged <6 years were at significantly lower risk for *O. viverrini* (OR = 0.12, p = 0.006) and hookworm infection (OR = 0.08, p = 0.015) compared to older age groups. In contrast, they had a significantly higher risk of being infected with *A. lumbricoides* than did other age groups (OR = 5.31, p = 0.035; Table 2).

**Table 2:** Summary results for logistic regression analysis for the association between risk factors and helminth infections (n = 574)

	***O. viverrini***		**Hookworm**		***T. trichiura***		***A. lumbricoides***	
	**OR (95% CI)**	**p-value**	**OR (95% CI)**	**p-value**	**OR (95% CI)**	**p-value**	**OR (95% CI)**	**p-value**
**Univariate logistic regression**								
Gender								
Male	1.67 (0.98–2.83)	0.057	1.45 (0.89–2.37)	0.128	1.52 (1.07–2.15)	0.020	0.68 (0.39–1.19)	0.178
Female	referent		1.00		1.00		1.00	
Age group (in year)								
<6	0.05 (0.01–0.36)	0.003	0.18 (0.05–0.67)	0.010	1.16 (0.53–2.54)	0.709	4.86 (1.05–22.56)	0.043
06 - 15	0.12 (0.01–0.89)	0.038	0.49 (0.14–1.76)	0.281	0.99 (0.49–2.01)	0.967	1.90 (0.41–8.78)	0.409
16 - 29	0.14 (0.02–1.08)	0.060	0.48 (0.13–1.71)	0.256	0.78 (0.38–1.63)	0.518	2.18 (0.47–10.14)	0.322
30 - 39	0.18 (0.02–1.51)	0.115	0.91 (0.22–3.71)	0.896	0.77 (0.36–1.68)	0.518	1.69 (0.33–8.49)	0.525
40 - 49	1.00		0.47 (0.12–1.88)	0.286	1.14 (0.50–2.59)	0.753	1.92 (0.36–10.47)	0.444
50 - 59	0.36 (0.03–4.10)	0.410	1.00		1.00		1.00	
60+	1.00		2.17 (0.21–21.9)	0.510	0.57 (0.19–1.63)	0.293	2.28 (0.36–14.54)	0.384
Ethnicity								
Laoloum	2.28 (1.33–3.91)	0.003	0.57 (0.35–0.94)	0.029	0.24 (0.17–0.35)	<0.001	0.28 (0.15–0.53)	<0.001
Laotheung	1.00		1.00		1.00		1.00	
Availability of latrine at home								
No	1.21 (0.63–2.32)	0.561	1.54 (0.86–2.75)	0.141	1.05 (0.66–1.67)	0.828	0.85 (0.42–1.70)	0.645
Yes	1.00		1.00		1.00		1.00	
**Multivariate logistic regression**								
Gender								
Male	1.90 (1.09–3.31)	0.023	1.43 (0.87–2.37)	0.159	1.43 (0.99–2.07)	0.06	0.59 (0.33–1.05)	0.074
Female	1.00		1.00		1.00		1.00	
Age group (in year)								
<6	0.12 (0.02–0.54)	0.006	0.08 (0.01–0.61)	0.015	n.a.		5.31 (1.12–25.18)	0.035
06 - 15	0.29 (0.06–1.31)	0.108	0.22 (0.03–1.69)	0.146			2.12 (0.45–9.97)	0.339
16 - 29	0.35 (0.08–1.64)	0.185	0.20 (0.03–1.62)	0.133			2.43 (0.51–11.54)	0.263
30 – 39	0.50 (0.10–2.49)	0.400	0.41 (0.05–3.51)	0.417			1.76 (0.34–9.02)	0.496
40 - 49	2.39 (0.21–27.58)	0.483	0.22 (0.03–1.87)	0.166			2.48 (0.45–13.77)	0.299
50 - 59	1.00		0.44 (0.04–4.46)	0.485			1.00	
60+	1.00						2.75 (0.42–18.04)	0.292
Ethnicity								
Laoloum	2.29 (1.31–4.01)	0.003	0.59 (0.35–0.99)	0.049	0.25 (0.17–0.36)	<0.001	0.27 (0.14–0.50)	<0.001
Laotheung	1.00		1.00		1.00		1.00	
Availability of latrine at home								
No	n.a.		1.37 (0.74–2.54)	0.318	n.a.		n.a.	
Yes			1.00					

With regards to knowledge of helminth infection and its risk factors, the following variables were retained as predictors for an *O. viverrini* infection in the multivariable logistic regression model: having heard about *O. viverrini*, eating raw fish salad, eating raw fermented fish and eating sour rice-fermented fish. Results showed that only eating raw fish salad was associated with the *O. viverrini* infection: those who reported eating raw fish salad *(Koi Pa, Lap Pa Nuew)* had a highly increased risk for infection compared to those who did not eat raw fish salad (OR = 16.1, p = 0.026).

### Knowledge, attitudes and practices of heads of households

Heads of households were interviewed about their knowledge of worm infection and prevention. Questions about liver fluke, hookworm, whipworm and roundworm were asked. Of the interviewees, 37.7% had heard about liver fluke before, named “*pha yat bai mai nai tap*” or “*san tap*” in Lao. A little more than half (59.2%) linked the infection to eating raw or insufficiently cooked fish dish, but only about a quarter of them linked it to the consumption of raw fermented fish sauce (24.5%) and raw sour rice-fermented fish (24.5%). Few household heads knew how to prevent liver fluke infections (14.3%). Among the STHs, roundworm was most well-known (70.8%). However, almost all of those people who knew of it (91.3%) attributed it to consumption of any type of raw food. Only a few people mentioned correct preventive measures (Table 3).

**Table 3:** Knowledge of helminthiases among heads of households in 10 villages in Saravane district, 2010 (n = 130)

**Item**	**Total**	**Male (n = 69)**	**Female (n = 61)**	**p-value**
**Knowledge about liver fluke**				
Heard about liver fluke	49 (37.7)	46.4	27.9	0.030
Transmission route for liver fluke infection:		**n = 32**	**n = 17**	
Eating raw fish dishes *(Koi Pa, Lap Pa Nuew)*	29 (59.2)	65.6	47.1	0.208
Eating raw sour fermented fish	12 (24.5)	18.7	35.3	0.200
Eating raw fermented fish	12 (24.5)	28.1	17.6	0.503
Eating pickled fish	6 (12.2)	12.5	11.8	1.000
Prevention of liver fluke infection:				0.374
Not eating any raw food: meat, shrimp etc.	21 (42.9)	40.6	47.1	
Avoiding raw fish consumption	7 (14.3)	18.7	5.9	
Maintaining good personal hygiene and use latrine	2 (4.1)	6.3	0	
Taking deworming medicine	2 (4.1)	3.1	5.9	
Avoiding cigarettes and alcohol	1 (2.0)	3.1	0	
Seeking health care	1 (2.0)	0	5.9	
**Knowledge about soil-transmitted helminths**				
Heard about hookworm	11 (8.5)	5.8	11.5	0.346
Transmission route for hookworm infection:		**n = 4**	**n = 7**	0.448
Eating raw meat, fish, vegetables	2 (18.2)	25.0	14.3	
Infection through food	1 (9.1)	25.0	0	
No idea	8 (72.7)	50.0	85.7	
Prevention of hookworm infection:				0.288
Taking deworming drugs	1 (9.1)	0	14.3	
Avoiding raw foodstuff consumption	3 (27.3)	50	14.3	
Maintaining good personal hygiene	2 (18.2)	25	0	
Heard about whipworm	2 (1.5)	1.4	1.6	1.000
Transmission route for whipworm infection	0	0	0	
Prevention of whipworm infection	0	0	0	
Heard about roundworm	92 (70.8)	69.6	72.1	0.748
Knowledge about transmission route:		**n = 48**	**n = 44**	
Hand washing before eating	8 (8.7)	10.4	6.8	0.716
Eating raw food	84 (91.3)	89.6	93.2	0.716
Prevention of roundworm infection:				
Hand washing before eating	19 (20.6)	27.1	13.6	0.111
Hand washing after using latrine	8 (8.7)	10.4	6.8	0.716
Taking deworming drug	11 (11.9)	8.3	15.9	0.341
Eating cooked food	33 (45.2)	58.9	41.2	0.129

Among household heads, 43.1% thought that hand washing before eating could prevent diseases such as abdominal pains and diarrhoea. Others (21.5%) did not see any link to health. A few household heads (2.3%) reported that in order to prevent worm infections, people should wash their hands before eating.

Only a few household heads (16.1%) reported having a latrine. Among them, females (92.3%) used them significantly more frequently than did male household heads (50.0%, p = 0.047; Table 4). About a quarter (26.1%) of household heads thought that the possession of a latrine would be convenient, as one would not get wet from rain and would not need to go to the forest. About a quarter (24.6%) of them believed that latrine possession could prevent disease transmission. Some heads of households (15.4%) understood that latrine use could prevent diarrhoea or even malaria.

**Table 4:** Risk behaviour of heads of households in Saravane district, 2010 (n = 130)

**Risky behaviour**	**Total**	**Male (n = 69)**	**Female (n = 61)**	**p-value**
Habit of eating raw food				
Eat raw fish dishes *(Koi Pa, Lap Pa Nuew)*	98 (75.4)	91.3	57.4	<0.001
Eat sour fermented fish *(Som Pa, Som Pa Noy/Som Pa Chom)*	59 (45.4)	56.5	32.8	0.007
Eat raw fermented fish *(Pa Daek, Pa Daek Nam)*	82 (68.1)	75.4	49.2	0.002
Eat raw sausages of pork *(Som Mou Dip)*	43 (33.1)	46.4	18.0	0.001
Eat raw beef salad *(Lap Xin Dip)*	93 (71.5)	88.4	52.5	<0.001
Eat raw vegetables	124 (95.4)	98.5	91.8	0.098
Cleaning vegetables before eating	123 (94.6)	98.5	90.2	0.051
Personal hygiene				
Hand washing before eating	127 (97.7)	97.1	98.4	0.663
Hand washing after defecation	108 (83.1)	81.2	85.2	0.535
Hand washing with soap	15 (11.5)	5.8	18.0	0.029
Wearing shoes when get outside/going to bush	119 (91.5)	89.9	93.4	0.540
Sanitation facility				
Having latrine at home	21 (16.1)	11.6	21.3	0.133
Using latrine every time	16 (76.2)	50.0	92.3	0.047
Using latrine sometime	4 (19.0)	37.5	7.7	
Not using latrine at all	1 (4.8)	12.5	0	

Raw food consumption was very common (Table 4). Among household heads, 75.4% reported regularly consuming raw fish dishes *(Koi Pa, Lap Pa Nuew),* 68.1% ate sour rice-fermented fish *(Som Pa, Som Pa Noy/Som Pa Chom)* and 45.4% ate fermented fish sauce *(Pa Deak, Pa Deak Nam)*. Many more men reported eating raw fish dish and other raw food dishes than did females (91.3% versus 57.4%*;* p < 0.001).

### Focus group discussion results

Among the 67 FGD participants, 50.7% reported having heard about liver fluke infection before; 43.3% associated the infection with eating any type of raw food and 4.5% linked the infection to eating raw vegetables; 11.9% related the consumption of raw or insufficiently cooked fish to a worm infection. Roundworm was the most well-known STH (77.6%; Table 5).

**Table 5:** Summary of focus group discussions, Saravane district, 2010 (n = 67)

**Items**	**n (%)**	**Male (n = 32)**	**Female (n = 35)**
Knowledge about worm infection:			
Heard about liver fluke *“san tap*” or “*pha yat bai mai nai tap*”	34 (50.7)	34.4	65.7
How to get infection with *O. viverrini*:			
Eating raw vegetables	3 (4.5)	3.1	5.7
Not maintaining good personal hygiene: hand washing, nail clipping, unclean environment	8 (11.9)	15.6	11.4
Eating any raw food: meat, snail, shrimp	29 (43.3)	34.4	51.4
Eating raw fish dishes *(Koi Pa, Lap Pa Nuew)*	8 (11.9)	12.5	11.4
Heard about roundworm	52 (77.6)	84.4	71.4
How to get infection with roundworm:			
It just occurs in our body	2 (2.9)	0	5.7
Eat any raw: meat, fish, shrimp, vegetables	7 (10.4)	15.6	5.7
Not maintaining good personal hygiene: not hand washing before eating, not clipping nails, flies touching our food, drinking dirty water	16 (23.9)	18.7	28.6
Heard about hookworm	3 (4.5)	6.2	2.8
Do not know	3 (4.5)	6.2	2.8
Heard about whipworm	2 (2.9)	6.2	0
Do not know	2 (2.9)	6.2	0
Heard about tapeworm	33 (49.3)	46.9	51.4
Eating raw food	8 (11.9)	21.9	2.8
Eating raw fish dishes	33 (49.3)	53.1	45.7
Perceptions of latrine use:			
Village will not be dirty	15 (22.4)	34.4	11.4
Flies do not touch our food anymore	4 (5.9)	12.5	0
Prevention of diseases/no transmission of diseases	10 (14.9)	18.8	11.4
Animals will not eat human faeces	6 (8.9)	0	17.1
Convenience (not getting wet from rain, convenience during the night and when sick)	13 (19.4)	6.2	31.4
Perceived liver fluke as health problem in community	6 (8.9)	12.5	5.7
Practices personal hygiene:			
Hand washing before eating	31 (46.3)	31.2	60.0
Saying that most of people in village do not wash hands with soap	10 (14.9)	0	28.6
Perceptions of personal hygiene:			
Hands will be clean if we wash hands before eating	10 (14.9)	0	28.6
We will be healthy if we wash hands before eating	3 (4.5)	9.4	0
We can not get any worm infections or diseases if we wash our hands before eating	20 (29.9)	28.1	31.4
Deworming in community:			
Getting dewormed	37 (55.2)	75.0	37.1
Treatment of tapeworm with traditional medicine	19 (28.3)	43.7	14.3
Treatment with modern medicine	18 (26.9)	31.2	22.8
Treatment of roundworm using modern medicine	2 (2.9)	2.9	0

Half of the participants (49.3%) who reported repeatedly eating raw fish dishes explained this issue. A man (aged 31 years) in Naphengyai village said that he loved to eat raw small cyprinoid fish salad, while two other men stated that eating raw fish dishes was their habit. One woman (aged 56 years) in Nongbouayai village said: “*We have the habit of eating raw fish dishes such as Koi Pa, Lap Pa Nuew, Koi Phan, Koi Cham because, on the one hand, we would like to change the style of cooking our usual dishes but, on the other hand, this type of dish like Lap Pa Nuew can be easily prepared and served for many people and is sufficient for all members in the family, even if we only could catch a small amount of fish*”. Eight women confirmed that women mostly prepared raw fish dishes. Some respondents (16.4%) reported consuming *Som Pa* (raw sour rice-fermented fish). Eight participants stated that this dish is rarely consumed.

The frequency of eating raw fish dishes depends on the availability of fish in the village. Three-quarters of participants (75.8%) reported that during the rainy season and thereafter, people more frequently eat raw fish dishes due to an abundance of fish in the ponds, rivers and rain-fed paddy fields. During the dry season, most people do not eat it more than once per month. A man from Chantai village (aged 45 years) stated that, “…*if fish were always available throughout the year, we would eat Koi Pa, Lap Pa Nuew, Koi Phan and Koi Cham every day*”.

When asked about personal hygiene, 46.3% mentioned that hand washing before eating was common. However, one woman (aged 41 years) mentioned that she sometimes forgot to wash her hands, particularly when very hungry after an intense period of working in rice fields. Ten participants said that no one in the village washes his/her hands after defecation.

When discussing the benefits of hand washing before eating, 29.9% of participants thought that it would not prevent any worm infections or any other diseases; 14.9% did it just to keep the hands clean; only a few participants (4.5%) linked it to health benefits.

Open defecation is very common and was mentioned by 16 of the 67 FGD participants (23.9%). When asked about latrine use, one man (aged 55 years) in Chantai village said that he had asked the government to support latrine construction because the surrounding forest is going to be *“finished”* soon, and they will not have a place to defecate anymore. Three men and one woman disliked the use of pit latrines due to their bad smell. Two men said: “*I feel uncomfortable if I use the toilet because I cannot get used to it. I am afraid all the time that others will open the toilet’s door”.* Another one said that, “…*what do we do if the toilet is full? I would say that to defecate in the forest is therefore a more convenient way*”.

During the discussion about the general benefits of latrines, only 14.9% participants thought that the use of latrines could prevent diseases from being transmitted in their village; four FGD participants thought that animals in the village would not eat human faeces any more if they had latrines. For instance, one woman aged 58 years from Naphengyai village said that, “…*animals would not eat human faeces and chickens would not die anymore…*”; the remaining seven participants mentioned that the villages would be cleaner and latrine use more convenient.

### Direct observation results

In two of the ten study villages, we could directly observe fish dish preparation during our field work. In Dongkoneua village, the village committee obtained the fish from the nearby village market and prepared a welcome meal for our team while we were working in their village. The food served during that meal was well cooked. In Chantai village, household members caught fish from a nearby river and prepared raw sticky fish salad *(Lap Pa Nuew)*. The team was told that *Lap Pa Nuew* must be served with raw fish.

Of ten study villages, five had at least a few latrines. However, in these five villages, fewer than 20% of households owned a latrine. Only one of four FGD villages (Naphengyai) had latrines. We looked at eleven latrines. Three had never been used before, while three others could only be used at night (i.e. they had no walls or roof and one was far from the source of water). Five latrines were used regularly (three were owned by village heads and two were owned by village health volunteers). We observed pigs, cows and buffalos straying freely in the study villages. Walking barefoot outside of the houses was very common, particularly for children.

## Discussion

This study deepened our understanding of helminth infections and multiparasitism in relation to knowledge, attitudes and practices in Southern Laos, where helminth infections are highly endemic. We conducted a cross-sectional survey in ten randomly selected villages in Saravane district. Stool examinations were performed on all members (aged ≥2 years) of selected households. At the same time, in-depth interviews with the heads of households, FGDs and direct observations were performed to better understand knowledge, attitudes and practices related to these infections.

Our findings show that trematode infections, i.e. *O. viverrini* infections, were highly prevalent (88.7%). In fact, prevalence rates were one and a half time higher than indicated in a previous assessment in 2004 (58.5%) [23], which might be explained by the fact that no community control activities were conducted between the two assessments. The current study employed a rigorous diagnostic procedure using two stool samples per person, which certainly contributes to explaining the higher prevalence rate of the current assessment. In addition, most individuals had multiple infections with two helminth species and a quarter was infected with more than three species. Co-infections were most frequent with hookworm, which was the second most frequent helminth infection found in our study (77.3%).

Our interview results reveal that household heads have very limited knowledge of *O. viverrini* infection, its route of transmission and potential means of prevention. The habit of eating raw and insufficiently cooked fish was a very common practice and open defecation was widespread. Nevertheless, some household heads recognised the benefits of latrine use and gave reasons for not using them. During the discussions, participants mentioned that they disliked using latrines because they were either not used to it or did not know what to do when latrines were filled up. Even among people who had a latrine, not all of them used latrines regularly. Interestingly, women used latrines significantly more than men, indicating that they see a much greater benefit from having latrines in a household. Direct observations fully supported this finding. Hence, future sanitation promotion community interventions should particularly focus on women in target communities.

Our triangulated methodological approach (i.e. stool examination, interview with heads of households, FGDs with community members and direct observation) allowed us to confirm findings from different sources of data and, hence, show the strong relationship between the high prevalence rate of helminth infections and the corresponding low level of awareness and knowledge of selected community members. However, our helminth infection assessment was performed with a Kato-Katz technique, which does not allow one to distinguish between *O. viverrini* and minute intestinal flukes (MIF) [26]. The most recent research based on purging infected individuals and molecular diagnostic tools showed that in Southern Laos, a considerable amount of MIF is present [27,28]. Thus, it is likely that the *O. viverrini* infection rate is over-reported in the current study. However, the route of transmission and preventive measures for these parasites are the same. Therefore, our findings of low community knowledge and awareness of worm infections is still valid.

Our research shows a distinct link between the prevalence of hookworm infection and the household heads’ knowledge of hookworm infections. A very high hookworm infection rate (86.6%) was observed and increased with age, from 71.2% in the youngest age group to 96.7% in the oldest age group. Conversely, household heads showed an extremely low level of knowledge of hookworm, e.g. awareness of infection risk through direct contact with soil. The majority of household heads reported wearing shoes outside but investigators directly observed that walking barefoot outside was very common in all villages. In addition, animal excreta originating from free straying animals such as pigs, cows and buffalos were observed in all surveyed villages. A recent study conducted in rural areas of central Thailand confirmed that walking barefoot outside and keeping livestock such as buffalos around the house were risk factors for hookworm infection [29]. Hookworm infection is known to be an important cause of iron deficiency, affecting the physical and cognitive development of children [9]. Therefore, building awareness of the modes of transmission, the relationship between infection and disease, and preventative measures needs to be addressed in these affected communities.

The Lao government has made considerable efforts to control STHs in women at reproductive age, in children under five years of age and in primary school children by providing regular mass treatment with mebendazole twice a year (single oral dose 500 mg) [30]. Against this backdrop, it is most astonishing that hookworm infection prevalence remains high. A high re-infection rate is a plausible explanation. Adequate health education messages and alternative communication channels, for instance peer education of mothers and primary school children, could address these issues. Another likely reason for the persistently high hookworm infection rate is the use of mebendazole instead of albendazole. Mebendazole has shown very low curative effects in recent studies in Southeast Asia [31,32] and therefore, albendazole (single oral dose of 400 mg) should be used in deworming campaigns.

*O. viverrini* infection is acquired through consuming raw fish [23,33,34]. In our study, we found that household heads have poor knowledge of liver fluke infection, including its transmission mode and means of prevention. This low level of awareness is certainly one reason why raw fish eating practices in these communities are still prevalent and have not changed substantially since the last assessment made by Sayasone and colleagues in 2004 [23]. A study conducted in northeast Thailand, where *O. viverrini* infection rate is very high, identified viable metacercariae in raw fish dishes such as *Koi Pa* and *Som Pa*[35]. Therefore, a prerequisite of preventing *O. viverrini* infection in an efficient way is to decrease raw fish consumption. Awareness building is the first step in this process, using adequate health education messages and approaches.

The populous, rural communities of the Laoloum people show a very rich cultural life, with high and regular intensity of religious ceremonies and social occasions (e.g. life stage and agricultural events), where raw fish dishes are a prominent marker of commensality and underline the importance of ethnic and religious belonging [36]. In contrast, Laotheung communities — and many of them adhere to local religious beliefs — do not, in general, engage in the multitude of elaborate ceremonial practices, which include the mandatory consumption of raw or insufficiently cooked fish. Furthermore, the Laoloum people and their villages have a much better, and thus more frequent, access to raw fish and raw fish dishes, particularly at markets, food stalls, in shops and through street vendors, than do the Laotheung who tend to live in somewhat remote areas without well-developed road networks and easy access to urban centres. Direct observation revealed that environmental conditions also contribute to the increased *O. viverrini* infection rate among the riparian Laoloum people: they live mainly along big rivers and streams with a large fish stock; the abundance of freshwater fish leads to regular consumption of raw fish [36]. In contrast, Laotheung communities live in a mostly hilly and rugged physical environment and do not have this rich quantity and quality of fish in the creeks; consequently, they consume raw fish less frequently.

This study indicates that men have a higher risk of being infected with *O. viverrini* than women do. This quantitative result is consistent with the findings from FGDs where men discussed their preference for eating raw fish dishes such as *Koi Pa*, *Koi Pa Siew*, and *Lap Pa Nuew*. Eating raw fish dishes is their habit and they would eat this food every day if fish were always available. Furthermore, men, as social, economic, political and religious representatives of their households and kin, participate in many more public and official events in the community than do women, where raw or insufficiently cooked fish dishes are usually served and consumed in commensality. The consumption of raw fish dishes is part of one’s social obligations and integration in Laos [36]. A study by Strandgaard and colleagues in Vientiane province obtained similar findings where more men ate raw fish dishes than women did [37]. It is also known that in endemic liver fluke areas a slightly higher prevalence rate of *O. viverrini* infection was observed in men [38]. Moreover, we found that more men harboured different worm species than women did. However, previous assessment in this district by Sayasone and colleagues in 2004 did not show a difference of infection rates between genders [23]. Appropriate health messages regarding flukes and STHs need to address these gender differences and tackle men’s riskier consumption pattern.

Our research confirms previous results that *O. viverrini* infection prevalence increases with age [23,25,39]. Although our study found that children <6 years had a low risk of *O. viverrini* infection compared to other age groups, these children showed a much higher *O. viverrini* infection rate than found in a previous study in 2000 (72.6% versus 27.9%) [39]. In addition, the majority of *O. viverrini*-infected children aged 16 and under was co-infected with hookworm (74.7%). Regarding helminth infection, our study found that 49.2% of heads of households allowed their children to share any raw fish dish with adults once they could eat by themselves, particularly at age three (mentioned by 67.7%) and at age two (6.1%). This long-term exposure (from childhood) to *O. viverrini* infection poses serious health problems: *O. viverrini* is carcinogenic for cholangiocarcinoma [8], particularly when the infected person turns 35 years or older [32,40]. The interrelation of *O. viverrini* infection and age becomes a serious public health issue and requires an intergenerational (i.e. acting between children and their parents) and transgenerational approach (i.e. acting across multiple generations such as children and elderly people) to health education, for instance, targeting health messages and measures to each different age group.

Although Saravane province, like other provinces in Laos, benefits from rapid economic and infrastructural development, helminth infections in general and fluke infections in particular remain an important public health concern. To address it appropriately, a community-wide intervention must be initiated to ensure access to treatment and health education to increase knowledge of worm infections. Based on our findings, a combination of informal and formalised health education activities might be best suited to broaden local people’s awareness and to promote adoption of healthy practices related to helminth infections.

## Conclusions

This study discerned the relationship between helminth infection rates and quantity and the level of awareness of parasitic infections and its risk factors in endemic liver fluke areas in Saravane district. It highlights that helminth infection, particularly fluke and hookworm infections, imply a high burden, followed by *T. trichiura, Taenia* sp. and *A. lumbricoides*, notably in communities where multiple helminth infections exist. However, specific knowledge and awareness of helminth infections was very limited, particularly regarding the mode of transmission and means of prevention. Consumption of raw and insufficiently cooked fish was widely practiced because these fish dishes are deeply rooted in the local culture of food and nutrition, particularly among the Laoloum. We observed poor personal hygiene practices and unreliable village sanitation; only a few households have access to a latrine but not everybody who has a latrine uses it regularly. This study calls for local authorities and communities in Saravane district to integrate actions to address helminth infections by building awareness and strengthening knowledge about worm infections and practices related to these infections. Furthermore, this research adds to the much-needed arsenal of mixed quantitative and qualitative methodological approaches in helminth studies. Qualitative methods in combination with quantitative baseline studies of infection rates shed light on social determinants of helminth infection as well as on cultural processes and community and individual health practices in regard to helminth vulnerability and reveal significant relations between prevalence rates, reasons for infection and appropriate problem solving measures.

## Competing interests

The authors declare that they have no competing interests and that the sponsors had no role in the study design, data collection and analysis, decision to publish, or preparation of the manuscript.

## Authors’ contributions

KP and PO conceived the study idea, and designed and analysed the data, and interpreted results together with PvE. KP coordinated and conducted field work and drafted the manuscript; VX carried out data collection and sample collection; YV carried out laboratory work; KA had overall responsibility for data collection; PvE and PO revised the manuscript. All authors read and approved the final submission.
